# From What to Why, the Growing Need for a Focus Shift Toward Explainability of AI in Digital Pathology

**DOI:** 10.3389/fphys.2021.821217

**Published:** 2022-01-11

**Authors:** Samuel P. Border, Pinaki Sarder

**Affiliations:** Department of Pathology and Anatomical Sciences, SUNY Buffalo, Buffalo, NY, United States

**Keywords:** digital pathology, deep learning, artificial intelligence, explainability, interpretability, machine learning, image analysis

## Abstract

While it is impossible to deny the performance gains achieved through the incorporation of deep learning (DL) and other artificial intelligence (AI)-based techniques in pathology, minimal work has been done to answer the crucial question of why these algorithms predict what they predict. Tracing back classification decisions to specific input features allows for the quick identification of model bias as well as providing additional information toward understanding underlying biological mechanisms. In digital pathology, increasing the explainability of AI models would have the largest and most immediate impact for the image classification task. In this review, we detail some considerations that should be made in order to develop models with a focus on explainability.

## Introduction

In recent years, the use of artificial intelligence (AI) to classify, segment, and otherwise gain new understanding of medical data has experienced rapid growth. The incorporation of AI in histopathology has great potential, providing pathologists with the ability to quickly render diagnoses for patients in a reproducible, objective, and time-efficient manner. Recent technological advances including the growing popularity of histology slide digitization and accessibility of high-powered computational resources have given rise to a field now referred to as digital pathology (Al-Janabi et al., 2012; Bera et al., 2019; Niazi et al., 2019). While the field of digital pathology has benefited from the advances made in more general domains of AI, it is important to remember the unique considerations that must be made when attempting to understand biological mechanisms. Leveraging domain knowledge held by the medical community is crucial in the development of AI-powered frameworks with a far-reaching impact on patient outcomes.

One of the best areas to study the impact of explainability is for the task of histopathological image classification (Holzinger et al., 2017; Pocevičiūtė et al., 2020). In current practice, pathologists looking at biopsy images synthesize available information based on their decades of education and experience in order to make diagnostic decisions. If a pathologist is asked to explain what specifically influenced their decision, they are able to indicate specific areas of the slide that contain lesions, cellular characteristics, or staining intensity variations that they know are associated with a particular disease. This interpretation by pathologists is the “gold standard” of an explainable histology system. However, this kind of patient-pathologist consultation is a rare occurrence in current practice despite demonstrated patient interest, particularly in cases of life-changing diagnosis (Gutmann, 2003; Manek, 2012; Lapedis et al., 2019). By incorporating AI-driven pipelines into their workflow, pathologists can greatly increase both the efficiency of diagnoses and their quantitative support. Complex computational models designed to tackle uncertainty through continuous exposure to diverse sets of data and intensive pathologist involvement represent a growing area of personalized medicine. Integrating prior medical knowledge with modern data science is the fundamental goal of Explainable AI, a major focus of this review.

**Figure 1:**
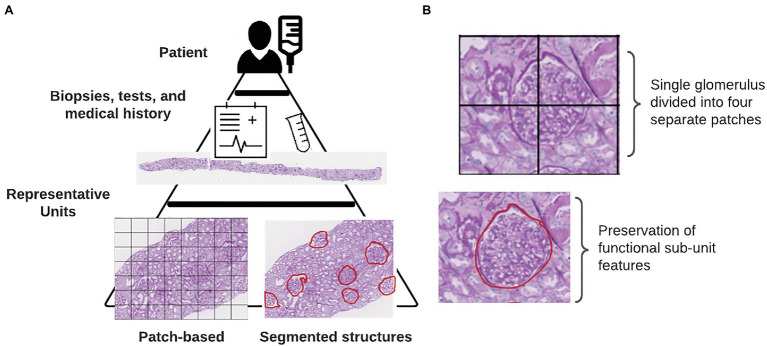
Defining representative units. **(A)** Internal hierarchy of medical data. Each tier represents an increasing complexity or resolution of underlying biological units. At the base of this pyramid is shown the two different methods for defining representative units in a particular study. **(B)** Consequences of using a patch-based instance definition include the partitioning of functional sub-units across multiple patches.

Explainable AI is far from a novel concept in the machine learning (ML) community (Goebel et al., 2018; Tosun et al., 2020a,b). While the presentation of new approaches for *post-hoc* explainers of deep convolutional neural networks (CNNs) is outside of the scope of this review, there are a few simple steps that can increase the interpretability and explainability of an AI-driven study (Figure 1). These steps include as: selection of representative units at appropriate scales, extracting quantitative features to discriminate informative units, and aggregating information on the whole slide image (WSI) level in order to generate patient-level conclusions.

The rest of this review will be organized following the above steps, with a focus on presenting the benefits and drawbacks of specific approaches in the current literature.

## Defining Representative Units

The inherent structure of data for medical ML tasks is hierarchical, consisting of multiple levels of resolution and detail (Figure 1). At the highest level, of course, is the patient. Within each patient, we have the results of tests, biopsies, and scans that give pathologists a look into the state of the patient’s health. In some cases, the results of genetic tests are also available which provide even finer scale information at the level of the DNA. Integrating information from lower-levels in order to make conclusions on the patient-level can be readily handled within a multi-instance learning (MIL) framework (Dietterich et al., 1997). The original example case of MIL given by Dietterich et al. describes a locked door for which there are several key rings available which might contain the correct key (Dietterich et al., 1997). Assuming the forgetful key master only knows which key rings contain keys that fit that door, we can use MIL to learn characteristics of keys on the positively labeled key rings from which predictions can be drawn for subsequent key rings. In the context of histology slides, WSIs from each patient are treated as the key rings (“bags” in MIL terminology) where the keys (“instances” in MIL terminology) are either individual pixels, patches with much smaller spatial dimensions than the full image, or annotated sub-structures within the image (Campanella et al., 2019; Hao et al., 2019; Diao et al., 2021; Lu et al., 2021). In digital pathology, how these instances are defined can markedly impact how the decisions made by a network can be interpreted in a biological context.

Unlike traditional image classification datasets like ImageNet or MS COCO, histology datasets contain images that are substantially larger in pixel dimensions (Deng et al., 2009; Lin et al., 2014). It is not unusual for WSIs to reach into the gigapixel dimensions, often with only a small fraction of input pixels containing tissue. Gilbertson et al. found that prior to employing JPG2000 compression, a WSI system could output as much as 2.7 GB of imaging data per square centimeter (Foran et al., 1997; Gilbertson et al., 2006). In order to enable DL-based analyses using data from WSIs, it is necessary to load each training image into the computer’s memory. Hardware memory constraints prevent the use of entire WSIs from being used as individual training examples. A common approach used by digital pathology researchers to circumvent memory limitations when using WSIs in conjunction with DL algorithms is to break up the image into patches of equal spatial dimensions. These patches typically contain sections of tissue between 40 and 250 kilopixels (0.01–0.625 mm^2^ with 0.5 μm/pixel resolution). While several existing works have achieved impressive performance treating these image patches as instances, we contend that the highest amount of explainability is obtained by instead using biologically relevant sub-compartments. Breaking the image into functional sub-units as opposed to arbitrarily assigned blocks has a better chance of conveying the biological relevance of each input object than when it is mixed in with other structures (Figure 1). Returning to the analogy of the key rings and the locked door, if our key ring contained thousands of keys, we can imagine that more is learned about what key will unlock the door when we focus on extracting features from each of these keys individually instead of the different parts of multiple keys grouped together.

Previous studies have been carried out with this principle in mind. Diao et al. trained a pair of CNNs to segment specific cell types and tissue regions from which they calculated quantitative features (Diao et al., 2021). This process allowed them to trace back their model predictions to specific cell or tissue types which allowed for simple localization of informative regions (Diao et al., 2021). Similarly, another study by Wang et al. developed a CNN to segment tumor regions from lung adenocarcinoma slides from which a set of 22 morphological features were used in order to predict survival probability (Wang et al., 2018). In both examples, CNNs were trained using pathologist annotations to efficiently generate datasets of specific cell and tissue types. By smartly selecting representative sub-compartments within large WSIs, model explainability is substantially increased in this study.

## Quantitative Feature Extraction

After selecting representative sub-units within a WSI, the next step in the pipeline should be to derive a way to compare these sub-units in order to assess the influence of treatment or disease in each of the provided groups. The decision of what type of features to extract from image data can have a substantial impact on the interpretability of the final results.

**Figure 2:**
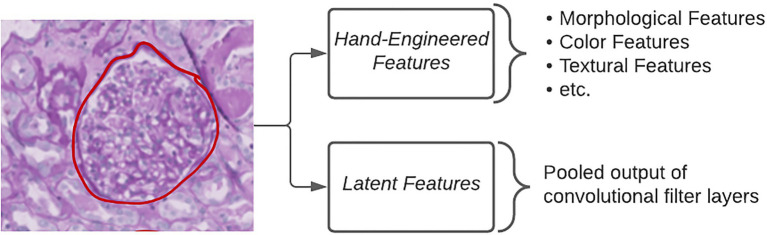
Extracting quantitative features. Different types of quantitative features extracted from images in order to make classifications using ML algorithms.

Standard DL approaches utilize latent features, defined as the pooled output of many convolutional filters, in order to classify images (Figure 2). The benefits of using this approach are that researchers are able to generate an arbitrarily large number of fine-grained features which have been shown to be highly discriminative. However, the way in which a computational model looks at image data and how a pathologist looks at image data differ immensely. Pathologists are trained to seek out particular lesions or cellular abnormalities that are known to be prognostic markers. When going through a WSI, pathologists record whether or not specific lesions were observed and at what frequency. Limitations with this kind of information include the requirement of an expert observer in order to properly catalog which results in a much larger amount of time needed per slide compared to fully computational methods. Furthermore, the semi-quantitative or qualitative nature of this kind of information can have a negative impact on inter-rater agreement.

The middle ground between the above two categories of features is referred to as hand-engineered features, which include sets of quantitative measures to describe the size, shape, texture, color, and proximity for given objects in images (Figure 2). These features can either directly relate to known morphological changes that are associated with disease, e.g., glomerular area in diabetic nephropathy, or indirectly examining qualitative attributes, such as the loss of mesangial matrix (mesangiolysis) through the calculation of several texture and color features. The specificity of hand-engineered features can also be modified to focus on sub-regions within each image through the use of additional segmentation methods. Color deconvolution, first proposed by Ruifrok et al. for the separation of immunohistochemically stained compartments, allows for the efficient segmentation of areas according to their biological properties (Ruifrok and Johnston, 2001). Studies, such as those by Yu et al. and Zhan et al., make use of an open-source software known as CellProfiler to quickly generate a large number of these hand-engineered features (Kamentsky et al., 2011; Yu et al., 2016; Zhang et al., 2019). CellProfiler provides the user with a wide variety of segmentation tools in a user-friendly interface for repeatable application to large image datasets (Kamentsky et al., 2011). More problem-specific feature extraction pipelines can incorporate existing domain knowledge as they condense the total amount of features to those that are known to be informative for that particular task (Ginley et al., 2019).

## Expanding Predictions to the Patient Level

After creating a quantitative understanding of features captured in each representative unit from a WSI, it now becomes necessary to understand the influence of each one of those units in the broader context of the patient. Modeling the contributions of each sub-unit on the final classification is a popular problem in the field of MIL and it is important that the manner in which instances are combined be interpretable to pathologists. Classical MIL techniques, such as Expectation Maximization-Diverse Density (EM-DD) and Axis-Parallel Rectangles, have demonstrated significant performance in defining bag-level distributions of data given feature values for weakly supervised tasks (Dietterich et al., 1997; Zhang and Goldman, 2001; Foulds and Frank, 2010; Carbonneau et al., 2018). Modern MIL approaches in digital pathology are designed to aggregate high dimensional features that are used by DL algorithms (Cosatto et al., 2013; Campanella et al., 2019; Sudharshan et al., 2019; Yao et al., 2020). Due to the stereological nature of renal biopsies, where it is not feasible to sample the entire kidney tissue, it is often the case where a small area of pixels contributes highly to the final WSI diagnosis. To best mimic this natural decision making procedure, some researchers have been incorporating recurrent neural networks (RNNs) and attention-based methods to iteratively learn to select informative regions from which patient-level conclusions are drawn. Campanella et al. employed an RNN that combined the influences of the most “suspicious” patches in order to render a diagnosis (Campanella et al., 2019). The most “suspicious” patches consisted of those with the highest ranked tumor probability by a prior CNN classifier. By using this method, authors were able to trace back their model’s diagnostic predictions to a subset of image regions containing patches with the highest probability of belonging to the tumor class. Ginley et al. also demonstrated the efficacy of an RNN to aggregate handcrafted feature values for renal glomeruli presented as a sequence within each renal biopsy (Ginley et al., 2019). For their work, they were more interested in determining the most influential hand-engineered features as opposed to most influential glomeruli, which they determined using a sequential dropout procedure for each feature to measure predictive value. Attention modules were incorporated into a CNN architecture by Ilse et al. in order to differentially weight input patch influences on image class prediction (Ilse et al., 2018). Integrating how patient-level conclusions are deduced from large input images ensures that the result is both accurate and interpretable.

## Incorporating Biological Interpretability

Strict criteria for network interpretability ensure that the model correctly assesses a candidate WSI based on etiologic features that can be interpreted by pathologists. To accomplish this, computational scientists must ensure that the networks they design are not only able to accurately diagnose biopsies, but also allow for the isolation and characterization of informative regions. This characterization process should account for the inherently hierarchical nature of medical data to allow for quick determination of important areas in the slide at multiple levels of magnification. By incorporating these considerations, computational networks can better mimic the “gold standard” of diagnostic explainability.

**Figure 3:**
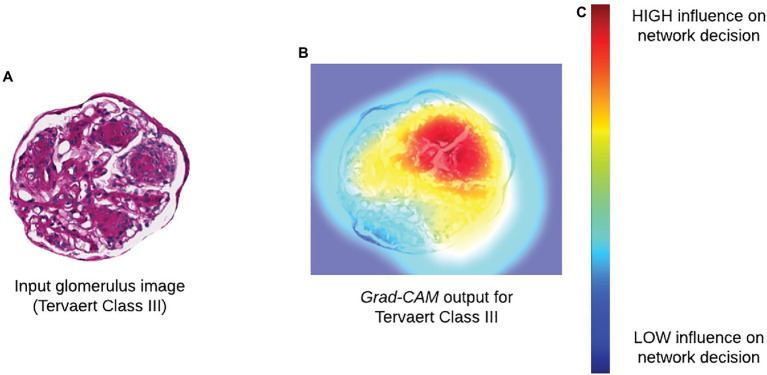
Incorporating biological interpretability. **(A)** Input glomerulus image to a CNN trained to predict severity of progression of diabetic nephropathy according to Tervaert criteria. **(B)** *Grad-CAM* output indicating relative influence of pixels in each region within the original image. **(C)** Colormap for *Grad-CAM* heatmap illustrating degree of influence of a particular region on the decision of a network.

Methods that seek to determine the focus of Neural Network (NN) models after training are referred to as “*post-hoc*” attention. This includes popular methods, such as saliency maps, deconvolutional networks, Grad-CAM, and DeepLIFT (Zeiler et al., 2010; Simonyan et al., 2013; Selvaraju et al., 2017; Shrikumar et al., 2017; Figure 3). While the internal operations vary, the output of each of these methods is a pixel-wise importance value for a specific classification output that is typically displayed as a heatmap overlaid on a tissue region. In addition to being a valuable tool for explaining the decisions made by a CNN, the authors of Grad-CAM also demonstrated how output heatmaps can be used as weak localization cues in a weakly supervised segmentation task (Selvaraju et al., 2017). When paired together with instance definition of functional sub-units, *post-hoc* techniques like Grad-CAM can be powerful tools in translating network predictions to approachable visual displays.

## Discussion

Throughout this review, we have assessed different works for their ability to provide users with sufficient levels of interpretability. In the field of digital pathology, interpretability is a critical feature of model design to ensure consistency and quality of patient treatment. Previous work by a mixture of institutions (academic, commercial, and regulatory) has highlighted concerns where AI algorithms have either introduced or mirrored systemic biases in their calculations (Minssen et al., 2020; Mehrabi et al., 2021). Without providing guidance to computational models that is based on prior knowledge, the model is forced to establish its own set of criteria that is not interpretable to a human observer. Through the incorporation of careful instance definition and hand-engineered features, the quality of algorithms using histopathological data can be elevated to the point that they are trusted for a greater range of applications. Model trust, reliability, and robustness require careful domain-specific considerations to be made so that data are appropriately processed to generate explainable results (Table 1).

**Table 1:** Glossary of terms.

Acronym used	Full expansion	Definition
DL	Deep Learning	A sub-field of Machine Learning and Artificial Intelligence where the final predicted value given an input is the result of the aggregation of information from many intermediary layers.
AI	Artificial Intelligence	A simulation of human intelligence by computers in order to solve complex problems.
ML	Machine Learning	Often used interchangeably with Artificial Intelligence, Machine Learning describes a set of algorithms wherein a computer learns to solve problems by analyzing input samples and their corresponding labels.
CNN	Convolutional Neural Network	A type of Machine Learning algorithm commonly used to classify images. Many image filters are compounded to extract information from images using convolution.
WSI	Whole Slide Image	A digitized image of a histology slide captured at full-resolution.
MIL	Multi-Instance Learning	A branch of Machine Learning dealing with data that is organized into groups.
EM-DD	Expectation Maximization-Diverse Density	A Multi-Instance Learning algorithm developed to extract characteristics of groups that best separate individual units into their respective groups.
RNN	Recurrent Neural Network	A type of Machine Learning algorithm commonly used to analyze sequences of data.
NN	Neural Network	A type of Machine Learning algorithm mimicking the flow of information between neurons in the brain.

## Author Contributions

SB contributed to the writing and figures for this manuscript. PS provided the guidance and editing. All authors contributed to the article and approved the submitted version.

## Funding

This project was supported by the NIH-NIDDK grant R01 DK114485 (PS), NIH-OD grant R01 DK114485 03S1 (PS), a glue grant (PS) from the NIH-NIDDK Kidney Precision Medicine Project grant U2C DK114886 (Contact: Dr. Jonathan Himmelfarb), a multi-disciplinary small team grant RSG201047.2 (PS) from the State University of New York, a pilot grant (PS) from the University of Buffalo’s Clinical and Translational Science Institute (CTSI) grant 3UL1TR00141206 S1 (Contact: Dr. Timothy Murphy), a DiaComp Pilot & Feasibility Project 21AU4180 (PS) with support from NIDDK Diabetic Complications Consortium grants U24 DK076169 and U24 DK115255 (Contact: Dr. Richard A. McIndoe), and NIH-OD grant U54 HL145608 (PS).

## Conflict of Interest

The authors declare that the research was conducted in the absence of any commercial or financial relationships that could be construed as a potential conflict of interest.

## Publisher’s Note

All claims expressed in this article are solely those of the authors and do not necessarily represent those of their affiliated organizations, or those of the publisher, the editors and the reviewers. Any product that may be evaluated in this article, or claim that may be made by its manufacturer, is not guaranteed or endorsed by the publisher.

## References

[ref1] Al-Janabi S. Huisman A. Van Diest P. J. (2012). Digital pathology: current status and future perspectives. Histopathology 61, 1–9. doi: 10.1111/j.1365-2559.2011.03814.x, PMID: 21477260

[ref2] Bera K. Schalper K. A. Rimm D. L. Velcheti V. Madabhushi A. (2019). Artificial intelligence in digital pathology - new tools for diagnosis and precision oncology. Nat. Rev. Clin. Oncol. 16, 703–715. doi: 10.1038/s41571-019-0252-y, PMID: 31399699 PMC6880861

[ref3] Campanella G. Hanna M. G. Geneslaw L. Miraflor A. Silva V. W. Busam K. J.. (2019). Clinical-grade computational pathology using weakly supervised deep learning on whole slide images. Nat. Med. 25, 1301–1309. doi: 10.1038/s41591-019-0508-1, PMID: 31308507 PMC7418463

[ref4] Carbonneau M.-A. Cheplygina V. Granger E. Gagnon G. (2018). Multiple instance learning: A survey of problem characteristics and applications. Pattern Recogn. 77, 329–353. doi: 10.1016/j.patcog.2017.10.009

[ref5] Cosatto E. Laquerre P.-F. Malon C. Graf H.-P. Saito A. Kiyuna T.. (2013). “Automated gastric cancer diagnosis on H&E-stained sections; ltraining a classifier on a large scale with multiple instance machine learning,” in Medical Imaging 2013: Digital Pathology: International Society for Optics and Photonics, 867605.

[ref6] Deng J. Dong W. Socher R. Li L.-J. Li K. Fei-Fei L. (2009). “Imagenet: A large-scale hierarchical image database.” in 2009 IEEE Conference on Computer Vision and Pattern Recognition; June 20-25, 2009; IEEE, 248–255.

[ref7] Diao J. A. Wang J. K. Chui W. F. Mountain V. Gullapally S. C. Srinivasan R.. (2021). Human-interpretable image features derived from densely mapped cancer pathology slides predict diverse molecular phenotypes. Nat. Commun. 12:1613. doi: 10.1038/s41467-021-21896-9, PMID: 33712588 PMC7955068

[ref8] Dietterich T. G. Lathrop R. H. Lozano-Pérez T. (1997). Solving the multiple instance problem with axis-parallel rectangles. Artif. Intell. 89, 31–71. doi: 10.1016/S0004-3702(96)00034-3

[ref9] Foran D. J. Meer P. P. Papathomas T. Marsic I. (1997). Compression guidelines for diagnostic telepathology. IEEE Trans. Inf. Technol. Biomed. 1, 55–60. doi: 10.1109/4233.594046, PMID: 11020810

[ref10] Foulds J. Frank E. (2010). A review of multi-instance learning assumptions. Knowl. Eng. Rev. 25, 1–25. doi: 10.1017/S026988890999035X

[ref11] Gilbertson J. R. Ho J. Anthony L. Jukic D. M. Yagi Y. Parwani A. V. (2006). Primary histologic diagnosis using automated whole slide imaging: a validation study. BMC Clin. Pathol. 6, 1–19. doi: 10.1186/1472-6890-6-4, PMID: 16643664 PMC1525169

[ref12] Ginley B. Lutnick B. Jen K.-Y. Fogo A. B. Jain S. Rosenberg A.. (2019). Computational segmentation and classification of diabetic glomerulosclerosis. J. Am. Soc. Nephrol. 30, 1953–1967. doi: 10.1681/ASN.2018121259, PMID: 31488606 PMC6779352

[ref13] Goebel R. Chander A. Holzinger K. Lecue F. Akata Z. Stumpf S.. (2018). “Explainable ai: the new 42?” in International Cross-domain Conference for Machine Learning and Knowledge Extraction; August 27-30, 2018; Springer, 295–303.

[ref14] Gutmann E. J. (2003). Pathologists and patients: can we talk? Mod. Pathol. 16, 515–518. doi: 10.1097/01.MP.0000068260.01286.AC 12748259

[ref15] Hao J. Kosaraju S. C. Tsaku N. Z. Song D. H. Kang M. (2019). “PAGE-Net: interpretable and integrative deep learning for survival analysis using histopathological images and genomic data,” in Pacific Symposium on Biocomputing 2020. eds. Altman R. B. Dunler A. K. Hunter L. Murray T. Klein T. E. (Singapore: World Scientific), 355–366. 31797610

[ref16] Holzinger A. Malle B. Kieseberg P. Roth P. M. Müller H. Reihs R.. (2017). Towards the augmented pathologist: challenges of explainable-ai in digital pathology. arXiv [Epub ahead of preprint].

[ref17] Ilse M. Tomczak J. Welling M. (2018). “Attention-based deep multiple instance learning.” in International Conference on Machine Learning; July 10-15, 2018; PMLR, 2127–2136.

[ref18] Kamentsky L. Jones T. R. Fraser A. Bray M.-A. Logan D. J. Madden K. L.. (2011). Improved structure, function and compatibility for CellProfiler: modular high-throughput image analysis software. Bioinformatics 27, 1179–1180. doi: 10.1093/bioinformatics/btr095, PMID: 21349861 PMC3072555

[ref19] Lapedis C. J. Horowitz J. K. Brown L. Tolle B. E. Smith L. B. Owens S. R. (2019). The patient-pathologist consultation program: A mixed-methods study of interest and motivations in cancer patients. Arch. Pathol. Lab. Med. 144, 490–496. doi: 10.5858/arpa.2019-0105-OA, PMID: 31429605

[ref20] Lin T.-Y. Maire M. Belongie S. Hays J. Perona P. Ramanan D.. (2014). “Microsoft coco: Common objects in context.” in European Conference on Computer Vision; September 6-12, 2014; Springer, 740–755.

[ref21] Lu M. Y. Williamson D. F. K. Chen T. Y. Chen R. J. Barbieri M. Mahmood F. (2021). Data-efficient and weakly supervised computational pathology on whole-slide images. Nat. Biomed. Eng. 5, 555–570. doi: 10.1038/s41551-020-00682-w, PMID: 33649564 PMC8711640

[ref22] Manek S. (2012). The pathology clinic–pathologists should see patients. Cytopathology 23, 146–149. doi: 10.1111/j.1365-2303.2012.00985.x, PMID: 22587554

[ref23] Mehrabi N. Morstatter F. Saxena N. Lerman K. Galstyan A. (2021). A survey on bias and fairness in machine learning. CSUR 54, 1–35. doi: 10.1145/3457607

[ref24] Minssen T. Gerke S. Aboy M. Price N. Cohen G. (2020). Regulatory responses to medical machine learning. J. Law Biosci. 7. doi: 10.1093/jlb/lsaa002, PMID: 34221415 PMC8248979

[ref25] Niazi M. K. K. Parwani A. V. Gurcan M. N. (2019). Digital pathology and artificial intelligence. Lancet Oncol. 20, e253–e261. doi: 10.1016/S1470-2045(19)30154-8 31044723 PMC8711251

[ref26] Pocevičiūtė M. Eilertsen G. Lundström C. (2020). “Artificial intelligence and machine learning for digital Pathology,” in survey of XAI in digital pathology. eds. A. Holzinger, R. Goebel, M. Mengel and H. Müller (United States: Springer), 56–88.

[ref27] Ruifrok A. C. Johnston D. A. (2001). Quantification of histochemical staining by color deconvolution. Anal. Quant. Cytol. Histol. 23, 291–299. PMID: 11531144

[ref28] Selvaraju R.R. Cogswell M. Das A. Vedantam R. Parikh D. Batra D. (2017). “Grad-cam: Visual explanations from deep networks via gradient-based localization.” in Proceedings of the IEEE International Conference on Computer Vision; October 22-29, 2017; 618–626.

[ref29] Shrikumar A. Greenside P. Kundaje A. (2017). “Learning important features through propagating activation differences.” in International Conference on Machine Learning; August 6-11, 2017; PMLR, 3145–3153.

[ref30] Simonyan K. Vedaldi A. Zisserman A. (2013). Deep inside convolutional networks: Visualising image classification models and saliency maps. arXiv [Epub ahead of preprint].

[ref31] Sudharshan P. J. Petitjean C. Spanhol F. Oliveira L. E. Heutte L. Honeine P. (2019). Multiple instance learning for histopathological breast cancer image classification. Expert Syst. Appl. 117, 103–111. doi: 10.1016/j.eswa.2018.09.049

[ref32] Tosun A. B. Pullara F. Becich M. J. Taylor D. L. Chennubhotla S. C. Fine J. L. (2020b). “Histomapr^™^: An explainable ai (xai) platform for computational pathology solutions,” in Artificial Intelligence and Machine Learning for Digital Pathology. eds. Holzinger A. Goebel R. Mengel M. Muller H. (United States: Springer), 204–227.

[ref33] Tosun A. B. Pullara F. Becich M. J. Taylor D. Fine J. L. Chennubhotla S. C. (2020a). Explainable AI (xAI) for anatomic pathology. Adv. Anat. Pathol. 27, 241–250. doi: 10.1097/PAP.0000000000000264, PMID: 32541594

[ref34] Wang S. Chen A. Yang L. Cai L. Xie Y. Fujimoto J.. (2018). Comprehensive analysis of lung cancer pathology images to discover tumor shape and boundary features that predict survival outcome. Sci. Rep. 8:10393. doi: 10.1038/s41598-018-27707-4, PMID: 29991684 PMC6039531

[ref35] Yao J. Zhu X. Jonnagaddala J. Hawkins N. Huang J. (2020). Whole slide images based cancer survival prediction using attention guided deep multiple instance learning networks. Med. Image Anal. 65:101789. doi: 10.1016/j.media.2020.101789, PMID: 32739769

[ref36] Yu K.-H. Zhang C. Berry G. J. Altman R. B. Ré C. Rubin D. L.. (2016). Predicting non-small cell lung cancer prognosis by fully automated microscopic pathology image features. Nat. Commun. 7:12474. doi: 10.1038/ncomms12474, PMID: 27527408 PMC4990706

[ref37] Zeiler M. D. Krishnan D. Taylor G. W. Fergus R. (2010). “Deconvolutional networks.” in 2010 IEEE Computer Society Conference on Computer Vision and Pattern Recognition; June 13-18, 2010; IEEE, 2528–2535.

[ref38] Zhang Z. Chen P. McGough M. Xing F. Wang C. Bui M.. (2019). Pathologist-level interpretable whole-slide cancer diagnosis with deep learning. Nat. Mach. Intelligence 1, 236–245. doi: 10.1038/s42256-019-0052-1

[ref39] Zhang Q. Goldman S. A. (2001). “EM-DD: An improved multiple-instance learning technique”, in Advances in neural Information Processing Systems, 1073–1080.

